# Cass County Medical Society

**Published:** 1886-04

**Authors:** R. L. McClung

**Affiliations:** Sec. and Treas. Cass Co. Med. Ass’n.


### CASS COUNTY MEDICAL ASSOCIATION.

To Daniel's Medical Journal, Austin.

*Dear Journal:*

THIS is the seventh year of our organization. We have done a good work in cementing the medical gentlemen of our section. We examine patients in the meeting and discuss the different methods of treatment. We exchange "Black

Lists," on the faith of Jim Bledsoe's creed, "They all put faith in his cussedness, and knowed he'd keep his word." Our first President was J. J. Davis, M. D. Our last is T. W. Connerly, M. D. We organized after the plan of the "American Medical Association." Success to legal medicine in Texas. Enclosed find two dollars for your JOURNAL, for our Association. Please send to yours respectfully,

R. L. McCLUNG, M. D.,
Sec. and Treas. Cass Co. Med. Ass'n.

